# Promote to protect: data-driven computational model of peer influence for vaccine perception

**DOI:** 10.1038/s41598-023-50756-3

**Published:** 2024-01-03

**Authors:** Sayantari Ghosh, Saumik Bhattacharya, Shagata Mukherjee, Sujoy Chakravarty

**Affiliations:** 1 Department of Physics, NIT Durgapur, Durgapur, India; 2 Department E and ECE, IIT Kharagpur, Kharagpur, India; 3 Centre for Social and Behaviour Change, Ashoka University, Delhi, India; 4 Behavioural Insights Unit of India, NITI Aayog, New Delhi, India; 5 School of Social Sciences, Jawaharlal Nehru University, New Delhi, India

**Keywords:** Computational science, Epidemiology, Psychology and behaviour, Socioeconomic scenarios

## Abstract

Vaccine hesitancy and acceptance, driven by social influence, is usually explored by most researchers using exhaustive survey-based studies, which investigate public preferences, fundamental values, beliefs, barriers, and drivers through closed or open-ended questionnaires. Commonly used simple statistical tools do not do justice to the richness of this data. Considering the gradual development of vaccine acceptance in a society driven by multiple local/global factors as a compartmental contagion process, we propose a novel methodology where drivers and barriers of these dynamics are detected from survey participants’ responses, instead of heuristic arguments. Applying rigorous natural language processing analysis to the survey responses of participants from India, who are from various socio-demographics, education, and perceptions, we identify and categorize the most important factors as well as interactions among people of different perspectives on COVID-19 vaccines. With a goal to achieve improvement in vaccine perception, we also analyze the resultant behavioral transitions through platforms of unsupervised machine learning and natural language processing to derive a compartmental contagion model from the data. Analysis of the model shows that positive peer influence plays a very important role and causes a bifurcation in the system that reflects threshold-sensitive dynamics.

## Introduction

The perceptions of a community about vaccines play a significant role in controlling and eradicating infectious diseases^1,2^. Vaccine hesitancy for the diseases that can be prevented using vaccines has been a concern for healthcare providers for quite some time now^3^. Vaccine hesitancy has been defined by the Sage Working Group on Vaccine Hesitancy and World Health Organization (WHO) as a “delay in acceptance or refusal of vaccines despite availability of vaccination services”^4^. In 2019, the World Health Organization has declared vaccination hesitancy as one of the top 10 obstacles for global health^5^. The extent of this hesitancy became evident during the last couple of years as the world was struggling with the COVID-19 pandemic caused by a virus called SARS-COV2. The COVID-19 pandemic has exacted a severe toll on individuals’ physical and mental health and lifestyle^6,7^. According to a report of the WHO on December 11, 2022, there were 6,658,277 deaths and 653,547,487 confirmed cases worldwide^8^. Since the start of the pandemic in December 2019, several pharmacological and non-pharmacological prevention strategies for COVID-19 have been tested with moderate benefits. Among the former, multiple vaccines against the virus have now been produced, and vaccines could be the best option to prevent the disease from spreading^9^.

Though several vaccines differing in composition, storage requirements, and effectiveness^10,11^ have been made available during the course of the pandemic, the acceptance of these vaccines was not immediate to many^12,13^. Overall, public response to vaccines is still poorly understood^14^. Moreover, though a small number of articles focus on levels of vaccine acceptance and its determinants with world-level cross-country studies^15,16^, there are very few attempts to understand and analyze vaccine hesitancy in the global south^12,13,17^. The COVID-19 vaccine uptake experience illustrates that even with no serious adverse effects reported from those vaccines, and with more virulent mutant strains being constantly identified, people’s inclination to get vaccinated may be affected by several biases. Across demography, age, and other socio-economic factors, there are different opinions, perceptions, and attitudes toward the vaccine, ranging from believing in the artificial origin of the virus^18^, lower perceived severity of COVID-19^19^ to low trust in vaccines and mistrust on the government^20^. Furthermore, exposure to misinformation regarding health consequences drives vaccine hesitancy, despite scientific evidence that may suggest otherwise^21^. A study on media from the global south conducted by^22^ finds that negative tweets on Twitter expressing poor vaccine confidence and misinformation received more public engagement and followers as compared to more favorable opinions.

The differences in beliefs and preferences of individuals and the societal health outcomes can trigger challenges for governments and public health experts. However, more recently, Chater and Loewenstein^23^ observe that what is often modeled as an individual-specific preference is not merely a reflection of an individual’s thought process. Rather, it is a consolidated output of several influencing factors that are systemic and related to peer groups and social networks. Accordingly, there have been attempts to understand and implement social dynamics in terms of elaborate quantitative equations and simulation rules. However, global factors like government policies, media campaigns, news broadcasts, etc., are not the only factors driving public attitude. Considering that human intent is not always driven by rational thoughts, an adopted attitude can get heavily influenced by the surrounding context and neighbourhood information. Discrete as well continuous opinion models, such as Sznajd model^24^, voter model^25^, majority rule model^26,27^, FJ model^28^, DeGroot model^29^, Ising model^30^ offer a solid set of tools to handle opinion formation in a society. On the other hand, information spreading models^31–34^ provide a compartmental depiction of the society to understand and predict the dynamics under various driving factors using Ordinary Differential Equations (ODEs). Growing drastically over the past decade, mathematical epidemiology models have been vastly applied to analyze the spread of information, rumor, habits, addiction, scientific ideas, opinions, petitions, etc.^35–39^, to successfully depict and understand these phenomena, and device strategies. Epidemic models were also tested in diverse socio-economic problems like development of consumer sentiment about economy, behaviour adoption related to capital market, early detection of financial bubbles, and prediction of stock buying/selling determinants^40–44^. In the context of COVID-19, extensive studies have been reported which detected *infodemics*, like spreading of misinformation, rumors, fake news, conspiracy ideas, and emotional state^45–47^. Even in the case of vaccine perception, people who are motivated by a desire for social empowerment may take a step towards forwarding their ideas among their peers with an urge to have an impact on others. Thus, the vaccine perception can propagate through the population like a beneficial virus causing a social contagion, making these dynamics a suitable candidate for employing a differential equation model.

Most mathematical models that study social contagion are formed through inputs based on straightforward observations and heuristic arguments. Thus, there is a likelihood that such model structures as well as their corresponding results may have a bias and may not directly reflect changing public perception accurately^48^. The question that we are interested in is the following: can we devise a methodology to derive these compartmental models from a survey data, and carry out mathematical analysis based on that model? Achieving this will provide us with a model which is ready to be analyzed with mathematical tools, but will be strongly rooted in the collected data of public opinion. Most of the literature in the area of public perception and policy making investigates preferences, values, beliefs, barriers, and drivers through closed or open-ended questionnaires^13,49,50^. While several survey-based studies attempt to understand the influence of people’s social networks on the adoption of a social policy, they commonly use statistical tools like percentage calculation, mean, median, and standard deviation (SD) estimation, etc., that do not do justice to the richness of the data. In this context, cutting-edge data mining tools using machine learning and natural language processing (NLP) can show us ways to extract deeper information from these data and quantify it. Furthermore, to the best of our knowledge, none of the social contagion studies use direct inputs from the survey data to design a data-driven model that reflects the decision dynamics. In a recent work, researchers have expressed their doubt about moral contagion models developed based on large-scale social media observations^51^, which confirms the necessity of taking a closer look at the methodically collected survey data. Thus, the key contribution of this paper to the literature is that it studies vaccine hesitancy behavior with a mathematical model that is based on solid foundations of survey data collection and analysis.

In this paper, we extract public opinion from open-ended survey answers through supervised machine learning and natural language processing tools with the goal to detect the reasons that can cause an attitude change, and map the detected transitions in the form of an Ordinary Differential Equation (ODE) model. Very recently, researchers^52^ have used NLP methods like Latent Dirichlet Allocation on a survey data to extract contagion-like effects in referral marketing. Effects related to peer influence and a misinformation epidemic have also been explored on COVID-19 vaccination intent by several researchers^21,53^, but surveys on vaccine perception and hesitancy have never been explored so far with a data-guided ODE contagion model. In this paper, we use a thoroughly designed, quantitative, mathematical contagion model with both positive and negative peer influence as well as prevalent global factors to explore these complex dynamics. Analyzing behavioral dynamics in terms of this ODE-based computational model that is derived directly from closed and open-ended answers of survey respondents helps us to devise behavioral strategies with a stronger quantitative understanding. To detect the reasons behind the changing vaccine perception of individuals, we extensively analyze the collected survey results in^53^.

Our methodology has been strategically designed to make sure that it goes beyond standard statistical analysis and speculative arguments for survey data analyses and mathematical model construction. We propose this novel methodology with an objective of threefold exploration:

- To analyze the survey responses by computational natural language processing algorithm to detect the behavioral barriers and drivers of positive/negative attitude towards the vaccine.
- To develop a differential equation-based model solely guided by the social compartments and behavioral transitions detected from data, and
- To study the results of the mathematical model formulated and quantify the effects of various factors on vaccination coverage for data-driven policy design.

**Figure 1:**
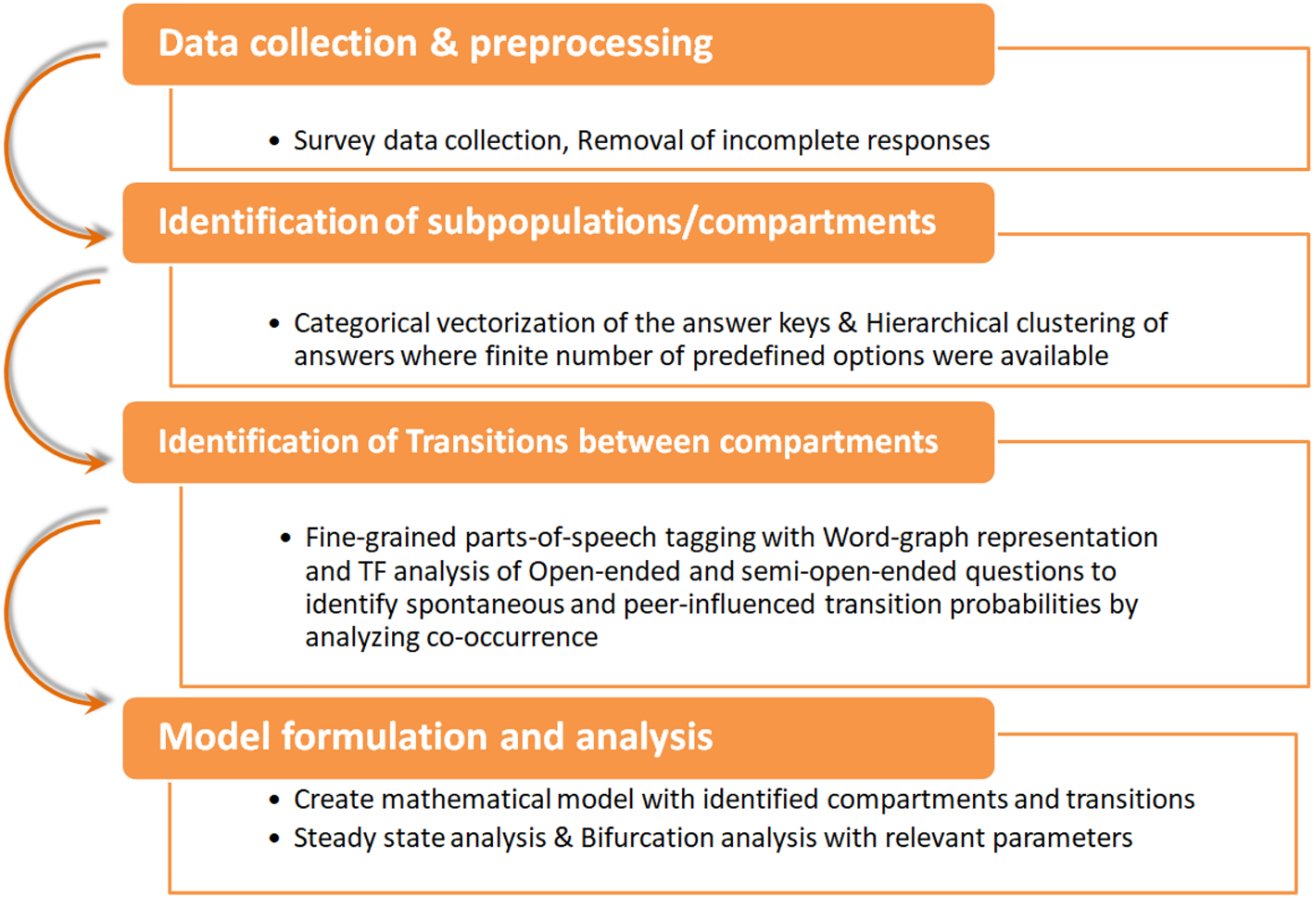
A block diagram of the proposed methodology.

The most significant strength of this novel methodology is that the model goes hand-in-hand with observed public attitudes towards vaccines. Finally, by combining open-ended survey answers with computational tools like, hierarchical clustering, word adjacency analysis, ODE modeling, and bifurcation analysis, we propose to devise a prescription for detecting the required conditions for the success of vaccination drive, overcoming vaccine hesitancy and misinformation. The main objective of the proposed methodology is to estimate a compartmental model from survey data that can depict the behavioral dynamics of a population. The estimated model is further analyzed to understand its behavior under different parametric conditions. The overall methodology pipeline has been shown in Fig. 1, which depicts that the entire methodology has four major interconnected stages: (1) data collection & preprocessing, (2) identification of subpopulation from data using an unsupervised learning technique, (3) identification of transitions between the compartments using part of speech tagging, and finally, (4) the analysis of the model to explore the underlying dynamics.

## Results

**Figure 2:**
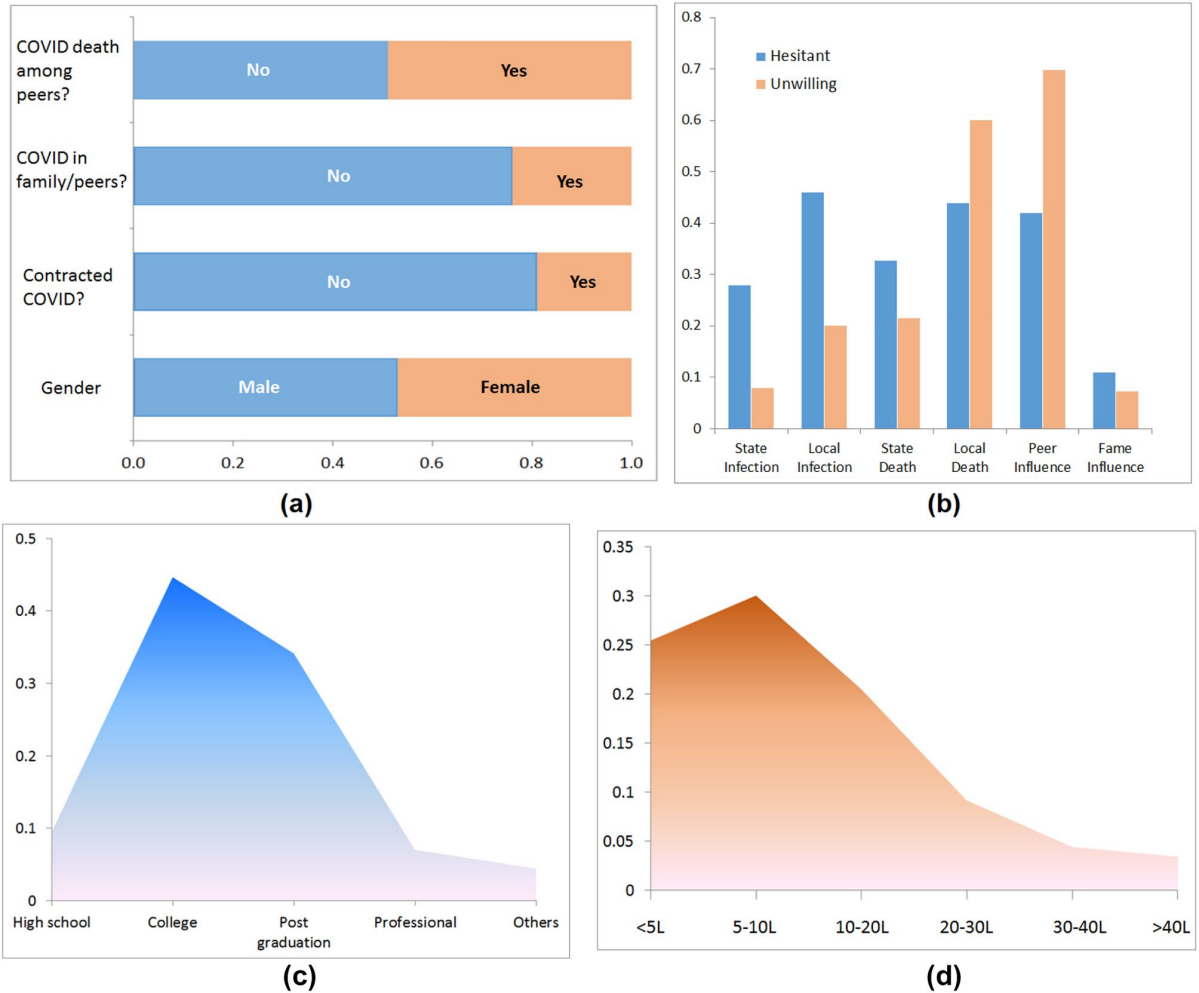
Some of the data statistics of the conducted survey: (**a**) some of the closed-end questions with binary options; (**b**) some of the probable reasons to change vaccine decisions; (**c**) educational background; and (**d**) annual income in rupees.

We analyze the survey data of Mukherjee et al.^53^ which addresses the issue of major drivers of COVID-19 vaccines in India through a questionnaire, consisting of both objective as well as elaborate, subjective answers. A total of 1196 respondents participated in the survey. PIN codes (equivalent to ZIP codes in the US) of respondents were collected to identify their geographic locations along with a time-stamp to associate responses with the extant reality of the epidemic in terms of caseload and deaths at their locations. It is reported that the data has good geographical variation as about 40% of the respondents are from major cities that are state capitals while 60% are from non-state capital cities, smaller towns, and villages. All respondents in the considered survey are between 18-76 years of age. The survey begins by asking questions like their genders, if they had contracted COVID-19 in the past, if there is any COVID infection or COVID-related death among their peers, etc. The responses to these questions are summarized in Fig. 2a. The respondents who were unwilling or hesitant to take the vaccine were asked about possible reasons that might change their decisions (Fig. 2b). The figure ahows that the hesitant or unwilling people were asked whether they would be willing to take the vaccine if the reported infection (in the state or local neighborhood), covid-related deaths (in the state or local neighborhood) increase. They were also asked about other reasons to affect their vaccine decisions, e.g., if celebrities promote vaccination (fame influence) or if their family and friends take the vaccine (peer influence). The Y-axis of Fig. 2b indicates the fraction of the subpopulation who would change their decisions. The educational background and the annual income of the respondents are also registered (Fig. 2c,d). As shown in Fig. 2b, peer influence is the most dominating factor that can change the decision of the respondents who were either unwilling or hesitant to take the vaccine.

In this work, we use Python on Jupyter Notebook along to analyze the data. The NLP part has been done using NLTK, spaCy and Gensim packages, whereas the graph construction and visualization have been done using ‘igraph’ package. The codes and the dataset can be downloaded from here: https://github.com/SaumikB/Survey. In this present study, we analyze the answers of these questionnaires using unsupervised machine learning and natural language processing tools to extract the underlying dynamical model that drives the vaccine acceptance behavior. Considering the cleaned and pre-processed data as the starting point, we focus on the closed and open-ended questions used for this survey. The final goal here is to construct a compartmental model for opinion diffusion. Any such compartmental model has two components: (1) *subpopulations or compartments,* consisting of people having different standpoints, to begin with, and (2) *transition probabilities*, i.e., the reasons and possibilities of inter-compartmental transitions, which could be either spontaneous or induced by another compartment. Once all the compartments and transitions are identified, a mathematical treatment can begin. Thus, for the identification of compartments, we proceed with hierarchical clustering of closed-ended questions of the survey, which can indicate clear differences in stand-points. Next, to identify the transitions, we analyze long answers with a close look to identify words that imply a transition and corresponding driving factors by word co-location. These major task has been consolidated in the *Step 2* and *Step 3* of Fig. 1 and elaborated below.

### Compartment identification: hierarchical clustering using unsupervised learning

**Figure 3:**
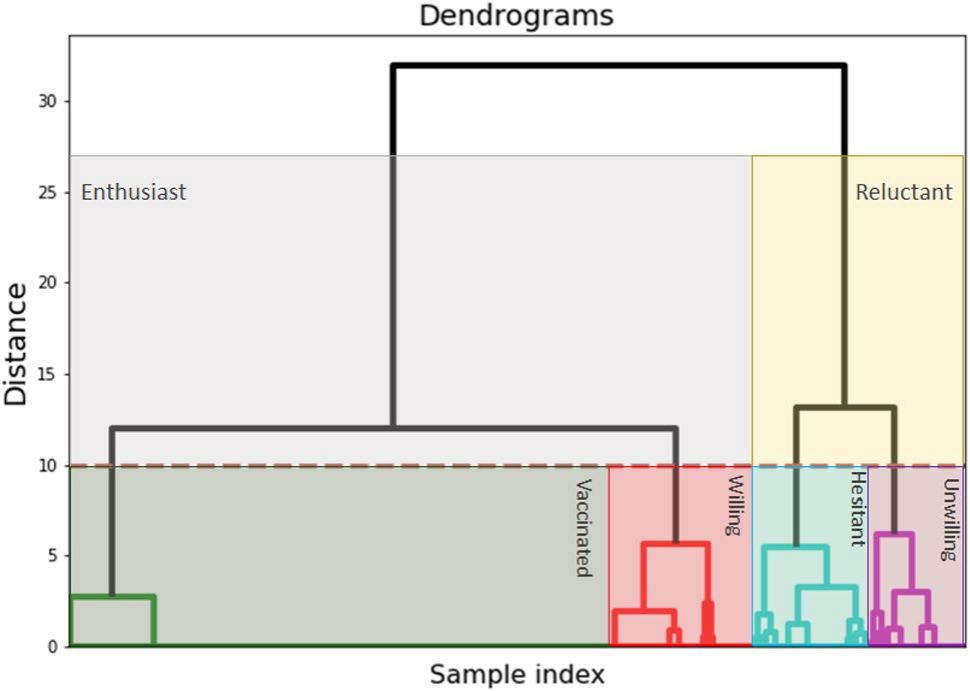
Detection of subpopulations from the close-ended questions using the hierarchical clustering. The dotted horizontal line depicts the threshold for detecting the subpopulations.

We draw conclusions about the model structure from data by analyzing eight questions for which a finite number of options are provided to select during the survey. These questions have predefined options (e.g., ‘Yes’, ‘No’, and ‘Maybe’) or a scale (with values 1-10) that can be selected by a respondent (*see* “Appendix” for details. Using categorical vectorization of the answer keys and the method of hierarchical clustering^52^, we identify four major clusters present in the participants, indicating the existence of four major subpopulations in the society ( Please refer to the Methodology Section). Thus, based on this observation, by establishing the existence of four major interacting compartments in the society in terms of vaccine hesitancy, the backbone model structure for the mathematical treatment gradually emerges out. The dendrogram in Fig. 3, representing the hierarchical clustering, shows one major bifurcation at the very beginning, which represents that, behaviorally, people are either enthusiastic about vaccination, or reluctant about it. Out of the final four clusters, (i) unwilling (*U*) and (ii) hesitant (*H*) clusters belong to the reluctant group, while (iii) willing persons (*W*), and (iv) vaccinated persons (*V*) are the vaccine enthusiasts. Substantial variability in these individual clusters was also detected in the hierarchical clustering, highlighting further complexity inside each of these subpopulations, indicating inhomogeneity in decision-making.

To dig deeper into this variability, we focus on the qualitative open-ended and semi-open-ended questions. Here, we refer to questions where long statements and explanations were encouraged to understand the reasons behind their answers, or where users could add answers other than the choices provided with the respective questions. As the dataset has around 1200 participants giving explanatory answers to 3-7 open-ended questions, instead of manual effort and heuristic arguments, we have employed term frequency analysis and fine-grained parts-of-speech tagging followed by word graph representation^54^, expecting to find rich, statistically significant conclusions. A series of data pre-processings (also mentioned in “Methodology”) are applied to the collected data to capture the user sentiments. Assuming the responses as a mixture of different sentiments and factors that control the perception of an individual towards vaccination, we performed the word tagging and word graph analysis on the pre-processed data. The analysis clearly shows that for decision-making, the participants considered different factors, which are either spontaneous or induced by peers. We elaborate on these results and formulate the data-driven compartmental model for vaccine perception (VP) dynamics in the next sections.

#### Identifying inter-compartment transitions: factors controlling vaccination decision

Through our rigorous analysis of the survey data, we arrive at some major conclusions regarding the driving factors of vaccination participation, which is a reflection of an individual’s perspective about the vaccine. We must pinpoint that these observations are arising from the outputs of NLP analysis of^53^ survey data.

*Transitions from Unwilling state*

For analyzing transitions from an Unwilling state, we focus on two open-ended questions:

- Q1: * “What are some major reasons for you to not want to take the vaccine?”*
- Q2:*“Is there any other reason which might make you change your decision? If yes, mention the reasons. If no, leave this blank.”*

**Figure 4:**
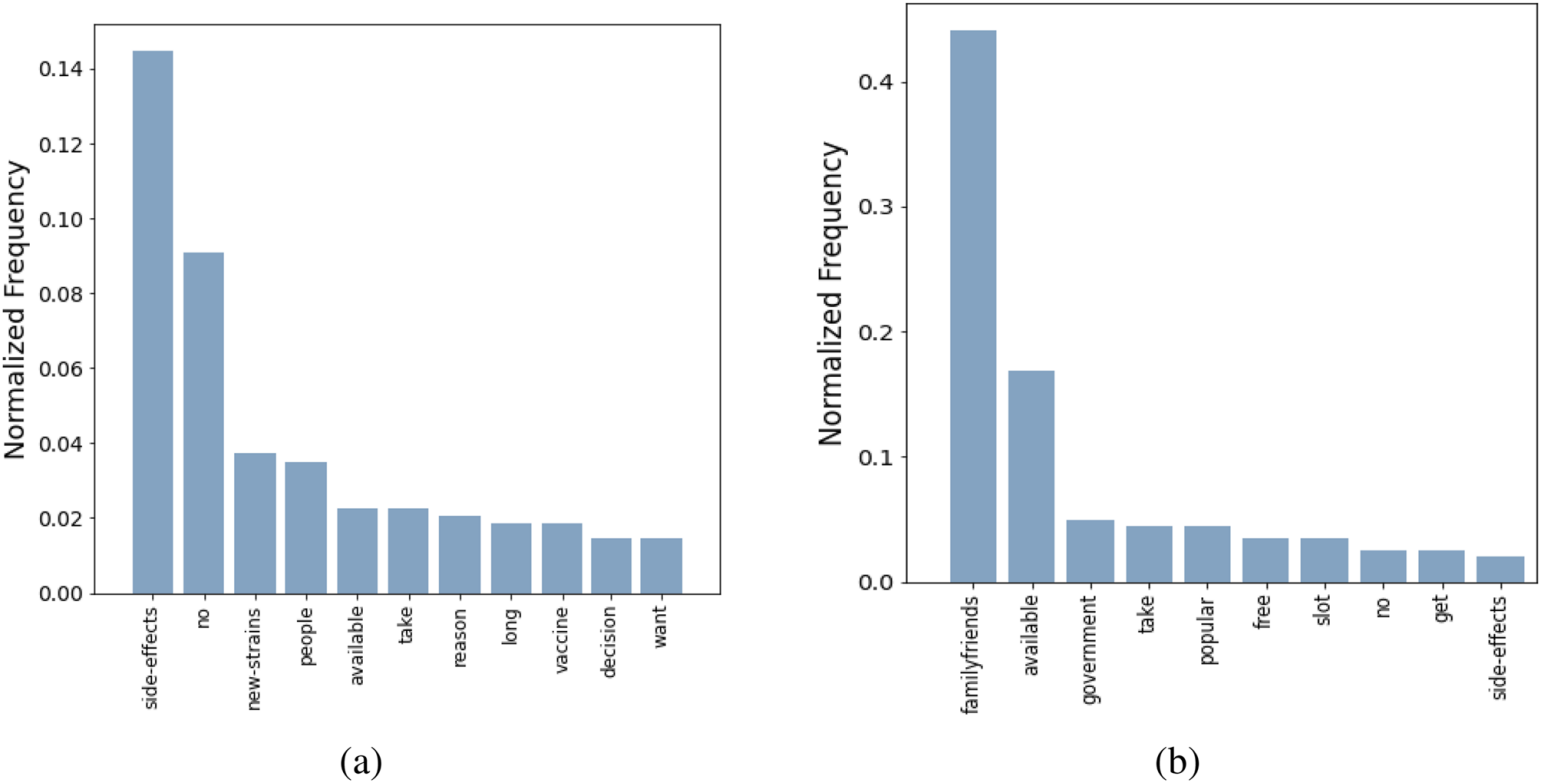
Word Histogram: Major reasons why (**a**) willing people may change their decisions and do not take the vaccine; (**b**) unwilling people may change their decisions and take the vaccine.

**Figure 5:**
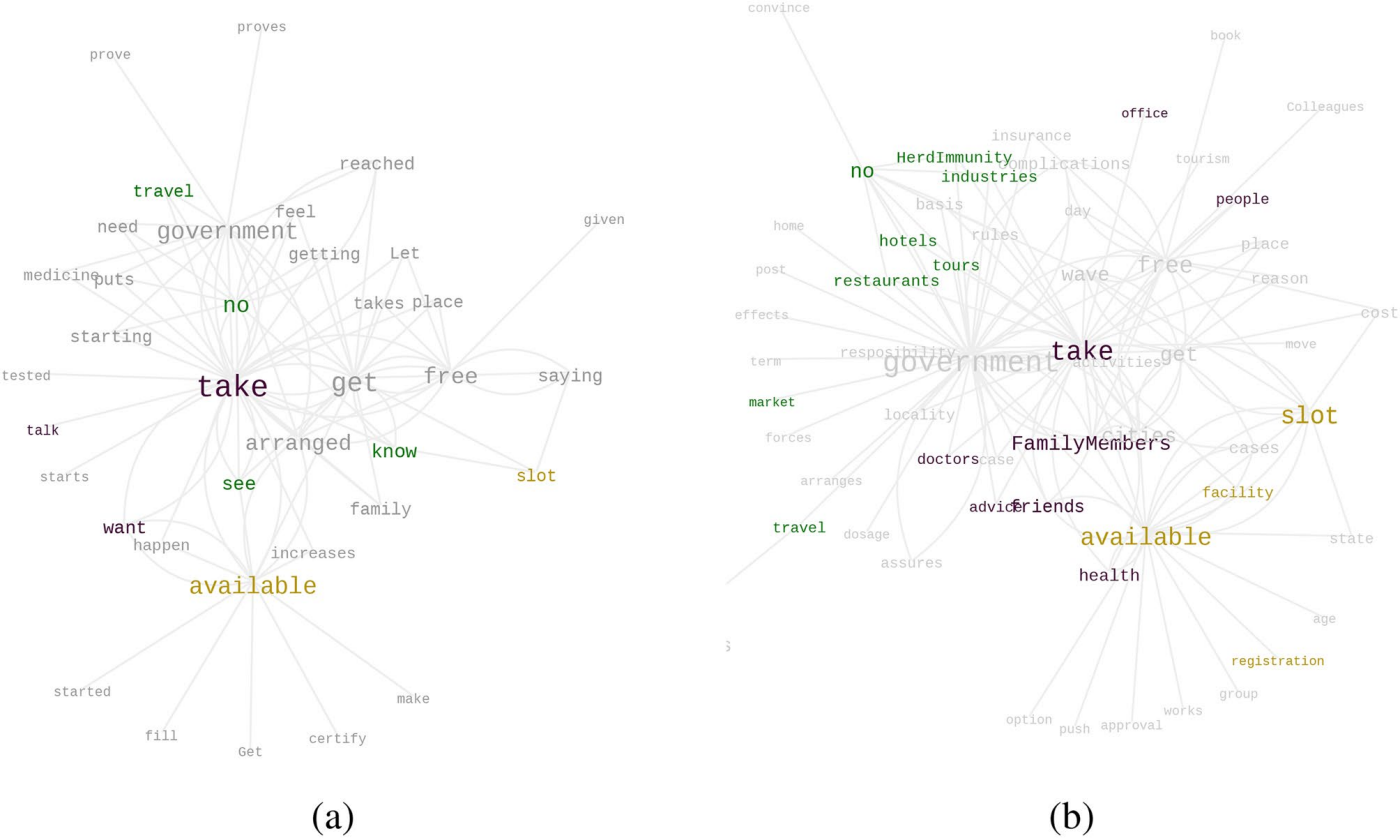
Driving factors to make the unwilling people partially interested to take the vaccine: (**a**) verbs associated with the top six keywords shown in Fig. 4b; (**b**) nouns associated with the top six keywords shown in Fig. 4b. Please zoom 300% for the best view.

Our goal here is to detect substantial reasons that may have caused the unwilling population to doubt their standpoint and make them switch to a less rigid, Hesitant (H) state. The word histogram associated with the answers is shown in Fig. 4b. Using parts-of-speech tagging and detecting word co-location, verbs and nouns adjacent to the top 6 words from the histogram are plotted in a graph representation in Fig. 5. In the figure, we note that the edges connect a major hub “take” to words like “FamilyMembers”, “Friends”, “people”, “colleagues”. Another notable edge connects “doctor − advice” which enforces the role of expert opinion. Moreover, frequent verbs like “assure”, “convince”, “talk”, “see” also indicate the importance of local inducers in the peer circles. It has also been observed in one of the direct questions that a substantial fraction of people (*refer* Fig. 2b) who identify themselves as unwilling to take the vaccine may switch their standpoint if a friend or family member takes the vaccine. All these observations together indicate the tremendous role of peer effect in U to H transition.

We further note words like “government” and “free” appear as global factors. A notable cluster can be found where the word “no” is connected with words like “hotel”, “travel”, “industries”, “market” etc. which indicates the concern of not being able to access these facilities without vaccination. Thus, we note that all these factors together may create a spontaneous but weak inclination for vaccination among unwilling people as well.

Thus, we observe possibilities of U to H transition, in both autonomous and persuaded ways, where interaction with vaccinated as well as willing people around them make the unwilling class to transit to the hesitant class, increasing their possibilities for a spontaneous vaccination over time. The consolidated results are shown in Table 1.

**Table 1:** Transitions from Unwilling subpopulation.

Transition Type	Reference Figure	Observed Word co-locations	Resultant transition	Induced By
Autonomous	Figure 5b	(no, HerdImmunity) (no, tours) (no, industries)	U $\rightarrow$ H	Self
Induced	Figure 5b	(FamilyMembers, take) (friends, take) (doctor, advice)	U $\rightarrow$ H	Willing vaccinated

#### Transitions from hesitant state

It has been observed that a non-trivial fraction of the survey participants is in a hesitant state. In the direct question of the survey^53^, it has been observed that around 13% of the total participants clearly mentioned that they are struggling with some confusion. There are two-way transitions possible from this population: Positive ($H \longrightarrow W$) or Negative ($H \longrightarrow U$). The relevant questions we focus on in this context are:

- Q1: *What are the reasons behind your hesitation?*
- Q2: *What might make you change (or not change) your decision if most people in the society take the vaccine?*

**Figure 6:**
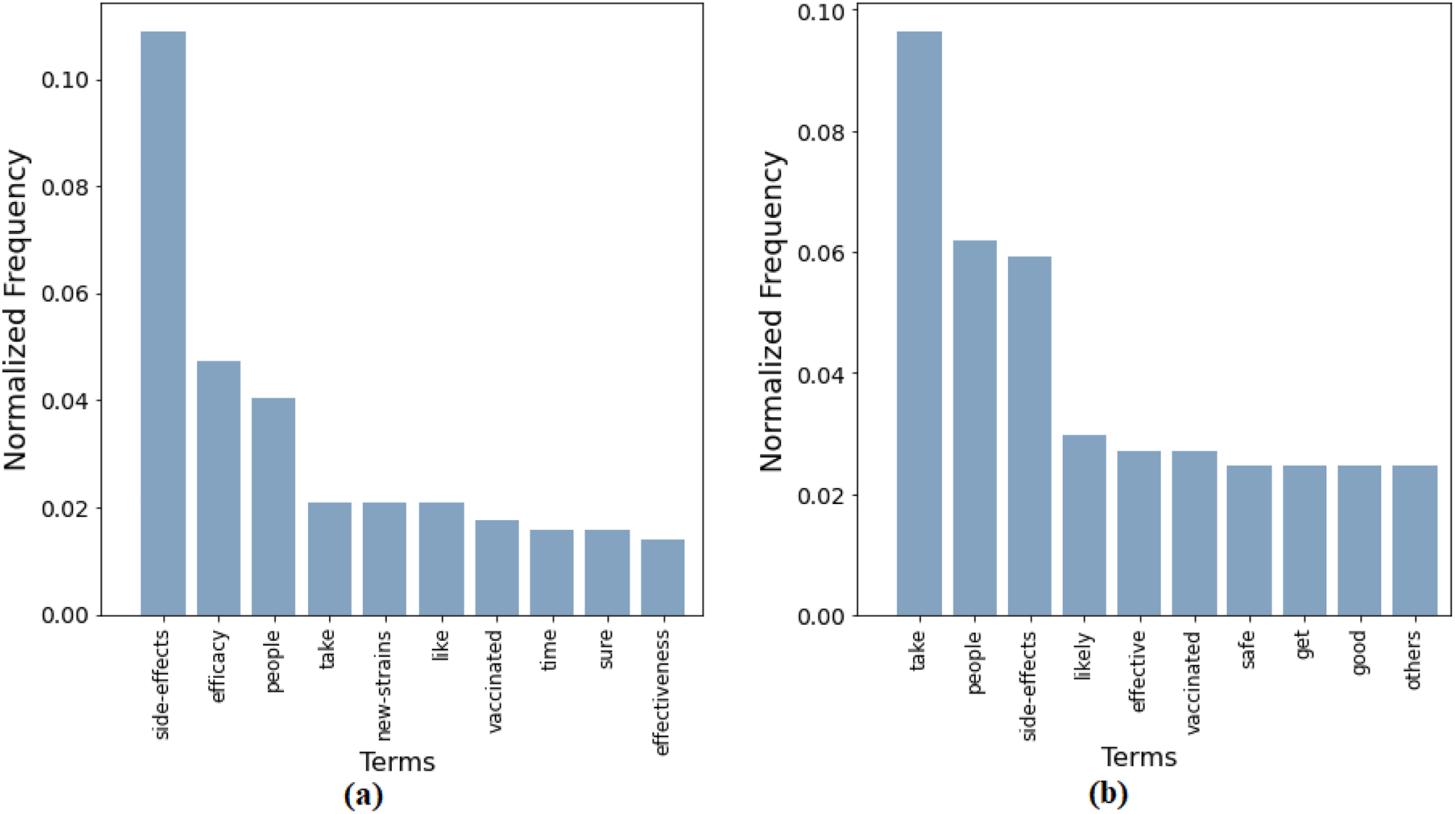
Word Histogram: (**a**) Reasons for being hesitant (H); (**b**) Reasons why hesitant people will change or not change their decision if most people in the society take the vaccine.

**Figure 7:**
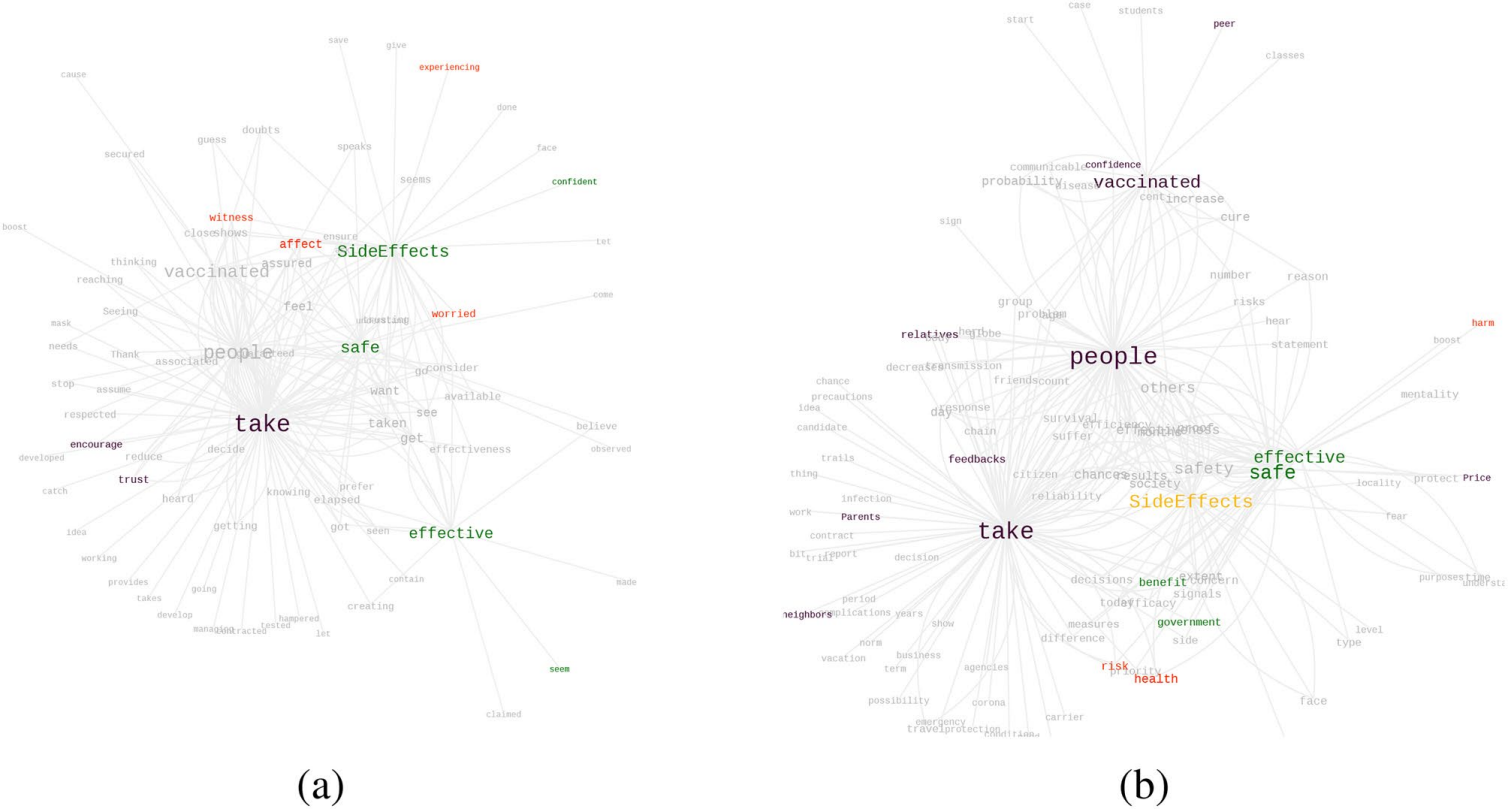
Driving factors to make the hesitant people to change their decisions: (**a**) verbs associated with the top six keywords shown in Fig. 6b; (**b**) nouns associated with the top six keywords shown in Fig. 6b. Please zoom 300% for the best view. The green (positive words) color and red (negative words) color show factors that are independent of peer influences; purple color words are factors associated with peer influence. Orange-colored word has positive or negative effects depending on context.

The major words emerging from term frequency are “SideEffects” and “Effective” (or “Efficiency”), which have been shown with the word histograms in Fig. 6. Word co-locations represented through graphs in Fig. 7 shows the word-hub “SideEffects” connected to “worried”, “health”, “harm” showing a spontaneous tendency of some hesitant people to shift towards an unwilling mentality. On the other hand, positive transitions show two distinct features: (i) Edges like “SideEffect−confident”, “feel−safe”, “seem−Effective” show inherent behavior shift towards vaccine participation, and (ii) edges like “Parent− takes”,“[vaccinated] people−effective”, “peer−vaccinated” shows that many people look for confirmation from peers before they decide to get vaccinated. The results are consolidated in Table 2. Here, the positive and negative words are identified using standard sentiment analysis word lists of positive and negative words. In the case of a neutral word, e.g., “health”, a manual marking is done.

#### Transitions from willing state

To understand the possible difficulties and concerns of the willing subpopulation, we analyze responses from the following question:

- *Is there any reason which might make you change your decision of taking the vaccine?*

**Figure 8:**
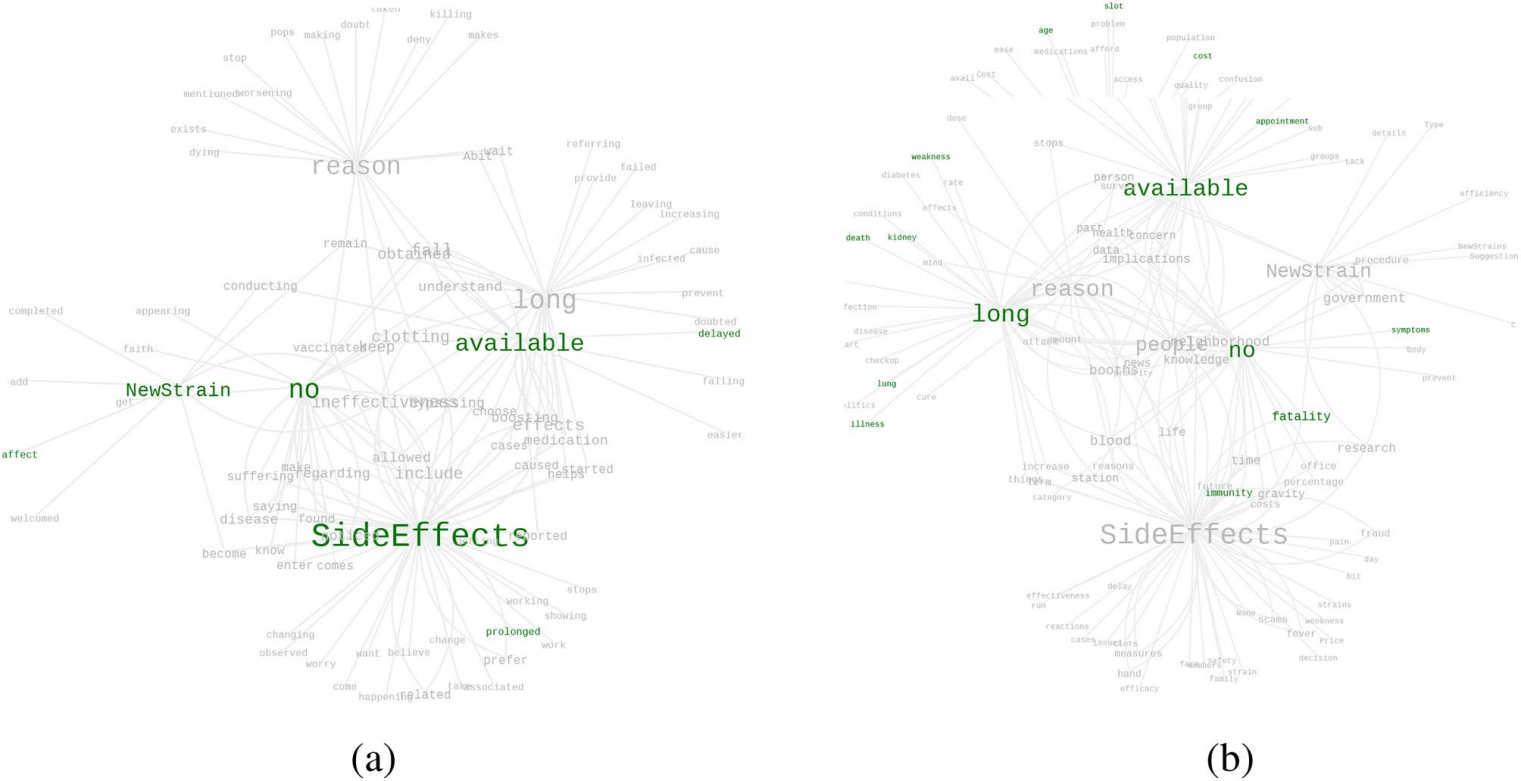
Driving factors to make the willing people hesitant to take the vaccine: (**a**) verbs associated with the top six keywords shown in Fig. 4a; (**b**) nouns associated with the top six keywords shown in Fig. 4a. Please zoom 300% for the best view.

The term frequency histogram is shown in Fig. 4a, and the graphically structured keywords are summarized in Fig. 8. We see that, after reaching the willing state, people are observed to face three major concerns, namely, fear of side effects and co-morbid conditions, reluctance due to inefficiency against new strains, and vaccine unavailability. The major hubs in the word graph are: “SideEffects”, “available”, “NewStrain” and “no”. Dominant connected words in the word graph are: “death”, “fatality”, “affect”, “efficiency”, “effectiveness” etc. Several words indicative of diseases, precise medical concerns, and existing co-morbid conditions are also observed: “kidney”, “lung”, “clotting”, “prolonged”, “long”, etc.

Interestingly, we also see words like “long”, “queue”, “slot”, “delayed”, “appointment”, etc. clustered together around “available”, indicating the issue of unavailability of vaccine or vaccination slots. It clearly indicates that the production bottleneck affected the participation in the vaccination drive. For the vaccinated subpopulation, in the direct questions, a percentage of participants indicated that they got vaccinated once they decided. However, we understand that though there was smooth access to vaccination for many, several people also suffered from availability issues. The observations are consolidated in Table 3.

**Table 2:** Transitions from Hesitant subpopulation.

Transition Type	Reference Figure	Observed Word co-locations	Resultant transition	Induced By
Autonomous	Figure 7a	(SideEffects, confident) (feel,safe) (seem,Effective)	H $\rightarrow$ W	Self
Induced	Figure 7b	(Parent, takes) ([vaccinated]people,effective) (peer,vaccinated)	H $\rightarrow$ W	Willing Vaccinated
Autonomous	Figure 7a,b	(SideEffects, worried) (SideEffects,health) (SideEffect,harm)	H $\rightarrow$ U	Self

**Table 3:** Transitions from Willing subpopulation.

Transition Type	Reference Figure	Observed Word co-locations	Resultant transition	Induced By
Induced	Figure 8a,b	(available, slot) (available, age) (available, appointment)	W $\rightarrow$ V	Vaccine availability
Autonomous	Figure 8a,b	(prolonged, SideEffects) (long, illness) (NewStrain, affect)	W $\rightarrow$ H	Self

### Derivation of the mathematical model from data analysis

**Figure 9:**
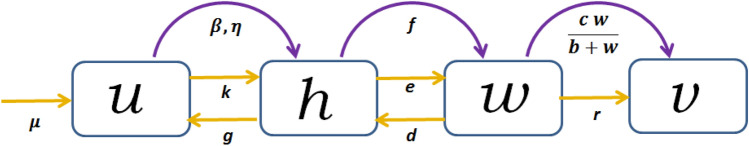
Data-driven compartmental model for vaccine perception. Boxes indicate the four interacting subpopulations. Purple arrows indicate induced transitions and orange arrows indicate autonomous transitions. Parameters associated with each transition are mentioned beside the respective arrows.

Based on the major observations in quantitative and qualitative analyses of survey data, we determine several subpopulations, transitions, and their possible causalities. Consolidating all these observations, we formulate the data-driven dynamical model as shown in Fig. 9. As indicated by our text clustering results, in our analysis, we consider four non-overlapping subpopulations *U*, *H*, *W*, and *V* such that the total population $T=U+H+W+V$. The normalized fractions of the total population in each of these compartments are denoted by lowercase characters: *u*, *h*, *w*, and *v*, where $u+h+w+v=1$. As reflected by Table 1*Row 2*, we assume that at a rate $\beta$ (and $\eta$), a willing (and vaccinated) person spreads the message regarding beneficial effects of the vaccine to a friend or family member from unwilling class, which may transform this unwilling person to someone who is hesitant (H), yet interested. People can also gather information related to the positive effects and necessity of vaccination from the problems they face due to shut down of industries and travel (Table 1*Row 1*). Through prevalent campaigns by government initiatives, print, broadcast, and social media, he may change his standpoint from unwilling to hesitant. Possibility of these spontaneous transitions has been incorporated into the model through parameter *k*.

Once in the hesitant population, we observed diverse confusion and dilemmas among the participants. As discussed in transitions from the hesitant state, both ways transitions are possible. People can become willing to take the vaccine, either through peer influence from vaccinated neighbors (with a rate *f*), who are well and can help them to overcome their fear of immediate side-effects, or by self-driven motivations (with a rate *e*) inspired from reasons like, safety, essential for travel and social gatherings (as per NLP results shown in *Row 1* and *Row 2* of Table 2). But, we also notice (as shown in Fig. 7), hesitant people can become unwilling again (rate parameter *g*) not only due to their fear of vaccines but also by understanding that they could be free riders and the system could reach herd immunity without them taking any risk (as shown in Table 2*Row 3*).

In the willing population, we still see the same possibilities of hesitation remaining, though on a small scale (shown in Fig. 8). Thus, a possibility for falling back to the hesitant state due to news of side-effects for people with prolonged illness and inefficiency for newer virus strains (Table 3, *Row 2*) is accommodated in terms of parameter *d*. But most of the people who are willing to get vaccinated will gradually get their vaccines. In India, two different vaccination drives were available. Health workers, doctors, police, and many government officials had priority to take the vaccine and had a constant supply of it. Thus, they never faced any crisis related to the vaccine supply, and once they became willing, they eventually got vaccinated. This transition is represented by ‘r’. Whereas for the common people, there was a reservation-based vaccination process. This indicates that at a moment, even if the number of willing people went very high, the rate of vaccination got saturated depending on the available vaccines in the public domain.. Thus, the question of a limited vaccine supply must be incorporated into the model. Especially for countries in the global south like India, it is impossible to supply vaccines for everyone willing in a short time span. As noted in Table 3, *Row 1*, the problem of unavailability of vaccines has been faced by several willing people. Thus, we incorporate the willing to vaccinated transition in both smooth (with enough availability, parameter *r*) as well as limited supply conditions. For putting the constraint of limiting supply, we take the transition rate to be $\Gamma (w)$, a nonlinear saturating function of *w*, which restricts the number of willing people who can get their vaccine shot per unit time. In our analysis, we take,

1 $$\begin{aligned} \Gamma (w)=\frac{cw}{b+w} \end{aligned}$$

This function implies that the number of vaccinated people can grow steadily when the willing fraction is small. However, as soon as the willing fraction is substantial, it gradually saturates, indicating a limit of maximum possible vaccinations per unit of time through that path.

We consider a constant birth and death rate, $\mu$, to assume a realistic scenario where people can join and leave the social system. Considering numbers of unwilling, hesitant, willing, and vaccinated individuals as continuously varying quantities, switching between subpopulations can be modeled by the following set of coupled ordinary differential equations:

2 $$\begin{aligned} u^{\prime}&= \mu - \beta u w-\eta u v+g h -(k+\mu ) u \nonumber \\ h^{\prime}&= \beta u w+\eta u v +k u+d w-f h v-(g+e+\mu ) h \nonumber \\ w^{\prime} &= f h v +e h-(r+d+\mu ) w-\frac{cw}{b+w} \nonumber \\ v^{\prime}&= \frac{cw}{b+w}+r w-\mu v \end{aligned}$$

All the transitions depicted in these equations are shown diagrammatically in Fig. 9 with the associated parameters mentioned.

### Equilibrium analysis of the model

Starting from survey responses of more than 1200 participants, we now have arrived at a set of coupled differential equations, depicting the dynamics. The proposed model is now open for mathematical treatment. As a preliminary investigation, it is interesting to analyze the system in a steady state. In steady state solution, we look for equilibrium solutions for a dynamical system in a long-time limit. For this analysis, we equate L.H.S. of Eq. 2 to zero and explore the steady state behavior of the system. Thus, we equate the rate of change of *u*, *h*, *w*, and *v* to zero. At this point, the system may have a low-vaccine equilibrium $V_0$, at which the majority of the population is unwilling. Also, the system may exhibit a vaccinated equilibrium $V^{\star }$ with a substantial percentage becoming vaccinated in the steady state. By setting the $u^{\prime}$, $h^{\prime}$, $w^{\prime}$ and $v^{\prime}$ of Eq. 2 to zero, all the components of $V^{\star }$ can be evaluated.

It is necessary to find out the stability of these equilibria to interpret the dynamics of the system. For analyzing the stability of the fixed points of our model, we have,

3 $$\begin{aligned} f_1&= \mu - \beta u w-\eta u v+g h -(k+\mu ) u \nonumber \\ f_2 &= \beta u w+\eta u v +k u+d w-f h v-(g+e+\mu ) h\nonumber \\ f_3 &= f h v +e h-(r+d+\mu ) w-\frac{cw}{b+w} \nonumber \\ f_4&= \frac{cw}{b+w}+r w-\mu v \end{aligned}$$

We obtain the Jacobian $J^\star$ (see “Methodology”) for all the steady states to analyze the eigenvalues and determine the stability of the steady states.

### Peer influence gives rise to bifurcation and bistability

**Figure 10:**
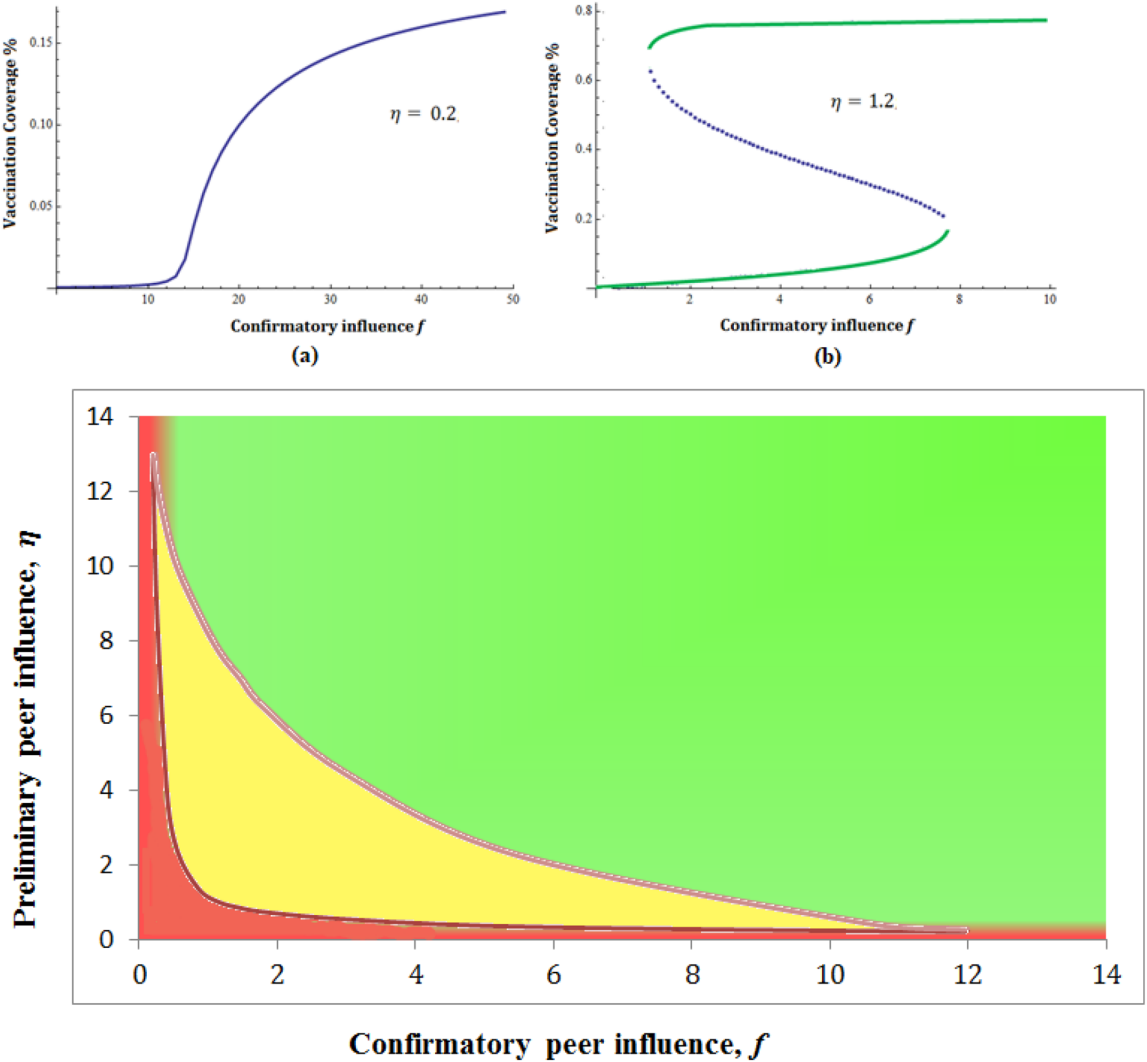
Effect of peer influence and Bistability: The figure exhibits a binary versus graded response dependent on the influence parameter $\eta$. Top panel: (**a**) For low values of $\eta$, a gradual increase in vaccination percentage is observed, which saturates at a certain percentage (13% for these parameter values). (**b**) For moderately high values of $\eta$, bistable dynamics can be seen. It shows that a sudden transition to a high vaccination percentage is possible beyond a threshold value of the confirmatory influence parameter, *f*. Other parameters are fixed at $\mu =0.2;e=0.01;r=0.2;g=0.5;d=0.05;b=0.1;c=10;k=0.01;\beta =2;$. Bottom panel: Phase diagram in $\eta -f$ plane, which indicates high and low vaccination state, marked in green and red color, respectively. In between these two monostable states, the region of bistability lies (marked in yellow).

Motivated by the findings of Mukherjee et al.^53^, we focus our investigation on peer influence parameters. The system shows two drastically different steady-state behaviors based on the parameters involved. In one regime of response, out of all possible solutions, only one, $V_0$, remains feasible and stable. An example parameter set would be $\mu =0.2$, $e=0.01$, $r=0.2$, $g=0.5$, $d=0.05$, $b=0.1$, $c=10$, $k=0.01$, $\beta =2$ and $\eta =0.2$. In this domain, as we observe the percentage of vaccination in Fig. 10a, with the increase in confirmatory peer influence parameter *f*, we observe a gradual slow response, where even for a very large value of *f*, only $15-20\%$ of vaccine coverage is achieved. More interestingly, however, we note the occurrence of saddle-node bifurcation for the other parameter regime with $\eta =1.2$. Here, both $V_0$ and $V^*$ remain feasible, and real solutions for a certain parameter space cause bistability to occur. Bistability is a phenomenon where there exists a choice for the system between two distinctly different outcomes. Here, both low and high vaccination coverage is possible when the system reaches equilibrium, depending upon the initial conditions. This history dependence is commonly known as hysteresis, drawing an analogy from the ferromagnetic systems.

In this second parameter regime, for lower values of peer influence parameters, $\eta$ and *f*, the vaccination coverage remains at a smaller level. However, as soon as these parameters cross a threshold, the system starts showing a bistable response. As shown in Fig. 10b, with the other parameters fixed, for peer influence $f=7.8, \eta =1.2$, a sudden transition to a high vaccination state occurs, giving rise to vaccine coverage of $78\%$ in contrary to a mere $19\%$. For any value higher than this threshold, the dynamics ensure substantial vaccination coverage. Interestingly, if now $\eta$ starts decreasing, the older threshold does not cause any transition to a lower vaccination level. The system remains at a high vaccination coverage state for a considerably lower range of $\eta$ values.

The results can be further examined using the two-dimensional phase space of $\eta -f$ plane (shown in Fig. 10, bottom panel). The red (green) color signifies a low (high) vaccination state. The intermediate yellow color denotes the region of bistability, where both the states are achievable, dependent on history, which gives the dynamics a robustness. The presence of bistability gives the system sustainability so that, once a transition occurs from the low to high vaccination state, the interactive dynamics inherently oppose any switch-back driven by the immediate fluctuations of the parameters; the whole social system works as a very robust switch, being unaffected by transient stochasticity. Thus, it can be concluded that for a high value of peer influence, the positive message remains in the system for a while, causing considerable advantage in vaccination coverage.

## Discussion

In this study, we analyze the perception and attitudes towards taking an existing vaccine and the key factors that drive the decisions of individuals. Through the lens of the proposed data-driven compartmental contagion model, we investigate the roles of local and global factors that influence this decision-making. The methodology designed to construct the model from data, is one of the major contributions of this paper. We used an unsupervised clustering algorithm to find the possible major sub-communities, followed by natural language analysis to find the key factors in the vaccine decision-making. We particularly focus on the open-ended questions from the survey data to design our data-driven compartmental model and the transitions present in it. In agreement with the survey findings, we also observe that peer influence is one of the key determinant factors. This gives the entire decision dynamics an inherent robustness which indicates that once a high vaccination state is achieved, a minor fluctuation in peer influence strength will not disturb the overall vaccination coverage. In other words, the system is analogous to a robust switch, which maintains its state even if the parameter fluctuates or reduces to a certain extent afterward. This result has important policy implications as such a strong influence of the local factor should be considered carefully while designing public health policies by governments.

We conclude by proposing the model and performing some preliminary analyses. Extensive mathematical treatment of the model, both in temporal and steady state scenarios will be addressed in a future work. One limitation of our study is that it does not fully incorporate the heterogeneity in the population. Thus, in an upcoming work, we intend to involve social network simulation of this contagion process along with the analyzed key factors to investigate the role of heterogeneity of a population, which is inherently present in any society. This additional level of complexity will make our observations closer to the real-world decision-making process flow. Moreover, further investigations, like the sensitivity of parameters, time solutions, etc., could be done for the mathematical model formulated in this work. Designing a knowledge-base from the actual survey data can also be a promising line of research that can provide deeper insights about the survey data, which can also be attempted in future studies.

## Methodology

### Data cleaning, preprocessing & hierarchical clustering

Each open-ended response of the survey data file was cleaned by standard NLP procedures, such as removing punctuation marks, removing URLs, lower casing, tokenization, stemming, and lemmatization, before we proceeded with our analysis. We received necessary ethical approval for the study from the Monk Prayogshala Institutional Review Board in India (IRB Approval Number:#076-021), and all methods were carried out in accordance with relevant guidelines and regulations. Our study uses data only from individuals who are at least 18 years of age, and informed consent was obtained from all respondents.

The subpopulations are identified using an agglomerative bottom-up hierarchical clustering algorithm. As the subpopulation structure is not known beforehand, we concatenate the close-formed responses of each respondent to a vector $\textbf{x}_i$, $i\in N_r$, where $N_r$ is the number of respondents, and initially, we consider each $\textbf{x}_i$ in a separate cluster $C_i$, $i\in N_r$. Then, we progressively combine the clusters with minimum pairwise average linkage measure $d_{al}(C_i,C_j)$ where $i\ne j$, until we have a single cluster. The average linkage measure $d_{al}(C_i,C_j)$ is defined as:

4 $$\begin{aligned} d_{al}(C_i,C_j)=\frac{1}{|C_i||C_j|}\sum _{\textbf{x}_1\in C_i, \textbf{x}_2\in C_j} d(\textbf{x}_1, \textbf{x}_2) \end{aligned}$$

where |.| denotes the cardinality of the set and $d(\textbf{x}_k, \textbf{x}_l)$ is a metric distance. In our work, we have considered $l_2$ norm as the metric distance. Finally, a threshold is selected where the distance metric is considerably large between two merged clusters (Please refer to Fig. 3). The only limitation of this method is that weak supervision is needed in case the clusters are very similar.

#### Fine-grained parts-of-speech tagging & Word-graph visualization

For $Q_o$ number of open-ended questions that are answered by $N_r$ respondents, we have collection of answers $\Theta =\{\theta _1,\theta _2\ldots \theta _i\ldots \theta _{Q_o}\}=\{\{\theta ^j\}_i\}$, $j\in N_r$, in the form of sentences for each open-ended question $q_i$, $i\in Q_o$. First, to identify the key factors that control the decision dynamics, we aim to create a bag-of-words $b_g^i$ that contains the most significant words that appeared in the answers for each question separately. To do so, we split each sentence $\theta ^j$ into words and combine them in a set of words $\phi _i$ considering all *j*. Next, we remove the stop-words^55^, like ‘the’, ‘do’, ‘is’, etc., from $\phi _i$ to find the actual significant words. The significance of a word is measured using the normalized frequency of occurrence of the word in $\phi _i$. We select the top-*k* number of most significant words from $\phi _i$ and create a bag-of-words $b_g^i$. Though $b_g^i$ reflects some important factors in decision-making, it is difficult to analyze such a set of isolated words. To further investigate a more meaningful interpretation of the answers $\theta _i$, we look into the association of other words with $b_g^i$ in the set $\theta _i$. We select each significant word $b\in b_g^i$ and separately analyze the associations of nouns and the verbs present in $\theta _i$ with *b*. The association is defined as follows. We define *b* and any word $\xi \in \{\{\theta ^j\}_i\}$ is associated (connected) if $\xi$ is either verb or a noun, and $\xi$ and *b* appears in the same sentence $\theta ^j$. Thus, for each question $q_i$, we get two word-association graphs ${\mathcal {G}}_n$ and ${\mathcal {G}}_v$ from the answer set $\theta _i$, where ${\mathcal {G}}_n$ and ${\mathcal {G}}_v$ indicate the graphs extracted from the associated nouns and verbs, respectively. The part-of-speech of each word in a sentence is identified using sentence tagging algorithm^54^.

#### Stability of equilibria

Let us consider a set of ordinary differential equations, ${\dot{\textbf{x}}}={\textbf{f}}(\textbf{x})$, with an equilibrium point ${\mathbf{x}}^{\star}$. We can linearize the equation by Taylor series expansion around the equilibrium as,

5 $$\begin{aligned} {\textbf{f}}(\textbf{x})={\textbf{f}}({\mathbf{x}}^{\star})+{\frac{\partial {\textbf{f}}}{\partial {\textbf{x}}}\Big |_{{\mathbf{x}}^{\star}}}(\textbf{x}- {\mathbf{x}}^{\star}) \end{aligned}$$

On the other hand, we can consider a small perturbation $\delta {\textbf{x}}$ from the steady state by letting, $\textbf{x}={\mathbf{x}}^{\star}+\delta {\textbf{x}}$. So, to study the behavior of $\delta {\textbf{x}}$ with time, we take a time derivative to find that

6 $$\begin{aligned} \delta \dot{\textbf{x}} = \dot{\textbf{x}}= \textbf{f}(\textbf{x}), \end{aligned}$$

as ${\mathbf{x}}^{\star}$ is a constant. Drawing the equivalence between Eqs. 5 and 6, as both express a form of $\textbf{f}(\textbf{x})$, we write,

7 $$\begin{aligned} \delta {\dot{\textbf{x}}}=J^{\star }\delta \textbf{x}, \end{aligned}$$

where $J^{\star }$ is the Jacobian evaluated at the equilibrium. For that equilibrium $\mathbf {x^\star }$ to be *stable*, *all* the eigenvalues of $J^\star$ should have *negative* real part.

### Supplementary Information

Supplementary Information.

## Data Availability

The datasets used and/or analyzed during the current study are available from the corresponding author upon reasonable request.
