# Supplementary material for: Promote to protect: data-driven computational model of peer influence for vaccine perception
Source: Sci Rep. 2024 Jan 3;14:306. doi: 10.1038/s41598-023-50756-3 (PMC10764860; doi:10.1038/s41598-023-50756-3)
Supplement: Supplementary file 1 — Supplementary Information. [file 41598_2023_50756_MOESM1_ESM.pdf]

# Appendix

**Academic Research Survey on COVID-19 in India**

Have you ever fallen sick with COVID-19?

| | |
|---|---|
| | Yes |
| | No |

How likely do you think you are to contract COVID-19?

| Not likely at all | 1 | 2 | 3 | 4 | 5 | 6 | 7 | 8 | 9 | 10 | Extremely likely |
|---|---|---|---|---|---|---|---|---|---|---|---|
| | | | | | | | | | | | |

Has anyone among your family members/relatives/friends/neighbours fallen sick COVID-19?*

| | |
|---|---|
| | Yes |
| | No |

Have you had any COVID-19 related death in your family/friends/neighbours/distant relatives? *

| | |
|---|---|
| | Yes |
| | No |

Are you willing to take the COVID-19 vaccine?

| | |
|---|---|
| | Yes |
| | No |
| | Maybe |
| | Already taken |

How certain are you about your decision about the vaccination?

| Not certain at all | 1 | 2 | 3 | 4 | 5 | 6 | 7 | 8 | 9 | 10 | Absolutely certain |
|---|---|---|---|---|---|---|---|---|---|---|---|
| | | | | | | | | | | | |

How effective do you feel the vaccines available in India are in preventing one from getting infected by COVID-19?

| Not effective at all | 1 | 2 | 3 | 4 | 5 | 6 | 7 | 8 | 9 | 10 | Completely effective |
|---|---|---|---|---|---|---|---|---|---|---|---|
| | | | | | | | | | | | |

Is there any reason which might make you change your decision of taking the vaccine? (If yes, mention the reasons such as side effects, costs, new strain of virus or any other reason that is important for you. If no, leave this blank.)

| |
|---|
| |

If you have not taken the vaccine then what are some major reasons for you to not want to take the vaccine? (Please give a detailed answer. You can mention if you have concerns regarding efficacy of the vaccine, side-effects, cost, new strain of virus or any other reason that is important for you)

| |
|---|
| |

If you have not taken the vaccine then will you consider taking the vaccine if any of the following happens? (click all that apply)

| | |
|---|---|
| | If the number of COVID-19 cases suddenly start increasing in your city or state but not necessarily in your neighbourhood |
| | If the number of COVID-19 cases suddenly start increasing in your neighbourhood |
| | If the number of COVID-19 related deaths start increasing in your city or state but not necessarily in your neighbourhood |
| | If the number of COVID-19 related deaths start increasing in your neighbourhood |
| | If you come to know that your immediate family members, friends, work colleagues/boss or family doctor have taken the vaccine. |
| | If you come to know that notable personalities like the chief minister, health minister or popular celebrities have taken the vaccine. |

Is there any other reason which might make you change your decision? If yes, mention the reasons. If no, leave this blank.

| |
|---|
| |

If you are undecided about taking the vaccine then what are the major reasons for your dilemma? (Please give a detailed answer. You can mention reasons like, efficacy of the vaccine, side-effects, cost, new strain of virus or any other reason that is important for you)

| |
|---|
| |

If most people in the society take the COVID-19 vaccine, will you be more likely to take it or less likely to take it yourself?

| | |
|---|---|
| | I will be more likely to take it myself |

| | |
|---|---|
| | I will be less likely to take it myself |

Why will you be more likely to take it yourself? (Please provide reasons)

| |
|---|
| |

Why will you be less likely to take it yourself? (Please provide reasons)

| |
|---|
| |

If you have not taken the vaccine then will you consider taking the vaccine if any of the following happens? (click all that apply)

| | |
|---|---|
| | If the number of COVID-19 cases suddenly start increasing in your city or state but not necessarily in your neighbourhood |
| | If the number of COVID-19 cases suddenly start increasing in your neighbourhood |
| | If the number of COVID-19 related deaths start increasing in your city or state but not necessarily in your neighbourhood |
| | If the number of COVID-19 related deaths start increasing in your neighbourhood |
| | If you come to know that your immediate family members, friends, work colleagues/boss or family doctor have taken the vaccine. |
| | If you come to know that notable personalities like the chief minister, health minister or popular celebrities have taken the vaccine. |

If you have taken the vaccine then please mention the date on which you got the first dose of the vaccine.

| |
|---|
| *MM/DD/YY* |

| |
|---|
| |

If you have taken the vaccine then please mention the date on which you got the second dose of the vaccine (Leave it blank if you have not taken the second dose yet).

| |
|---|
| *MM/DD/YY* |

Were you confused before you took the decision to take the vaccine? *

| | |
|---|---|
| | Yes |
| | No |

Reasons for your confusion (leave blank in case the answer to the previous question is 'No').

| |
|---|
| |

How much have you waited for your vaccine after you took the decision to take the vaccine?

| | |
|---|---|
| | Less than a week |
| | Less than a month |
| | 2-3 months |
| | More than 3 months |

What was the reason for the delay (if any) while taking the vaccine? It could be unavailability, price, further confusions or anything else.

| |
|---|
| |

| |
|---|
| |

How much do you agree or disagree with the following statement: 'Now that the COVID-19 vaccination drive has started in India, strict social distancing and mask wearing practices can be relaxed.'

| | |
|---|---|
| | Strongly agree |
| | Agree |
| | Not sure |
| | Disagree |
| | Strongly Disagree |

Why do you strongly agree? (Please give a few reasons for why you feel this way)

| |
|---|
| |

Why do you agree? (Please give a few reasons for why you feel this way)

| |
|---|
| |

Why are you not sure? (Please give a few reasons for why you feel this way)

| |
|---|
| |

Why do you disagree? (Please give a few reasons for why you feel this way)

| |
|---|
| |

Why do you strongly disagree? (Please give a few reasons for why you feel this way)

| |
|---|
| |

If you had a choice between gaining Rs. 500 for sure, and a lottery where we toss a fair coin and you have a gain of Rs. 0 with ½ chance [heads] and a gain of Rs. 1000 with ½ chance [tails], you would:

| | |
|---|---|
| | Choose the sure gain of 500 |
| | Choose the lottery |
| | Be indifferent between the two options |

If you had a choice between losing Rs. 500 for sure, and a lottery where we toss a fair coin and you lose Rs. 0 with ½ chance [heads] and a loss of Rs 1000 with ½ chance [tails], you would: *
If you had a choice between getting Rs. 2000 right now versus Rs. 4000 in six months' time, what would you choose? *

| | |
|---|---|
| | Choose the sure loss of 500 |
| | Choose the lottery |
| | Be indifferent between the two options |

If you had a choice between getting Rs. 2000 right now versus Rs. 4000 in six months' time, what would you choose?

| | |
|---|---|
| | Rs. 2000 right now |
| | Rs. 4000 in six months |

| | |
|---|---|
| | Be indifferent between the two options |

If you had a choice between getting Rs. 2000 in a year's time (12 months) versus Rs. 4000 in one year and six months (18 months) which would you choose? *

| | |
|---|---|
| | Rs. 2000 in one year's time |
| | Rs. 4000 in one year and six months' time |
| | Be indifferent between the two options |

What is your age?(in numbers)

| |
|---|
| |

What is your gender?

| | |
|---|---|
| | Male |
| | Female |
| | Other |

What is your educational qualification?

| | |
|---|---|
| | School - Standard 10+ |
| | School - Standard 12+ |
| | Graduation (B.A/B.Sc/B.Com/B. Tech etc.) |
| | Post Graduation (M.A/M.Sc/M.Tech/M. Phil/PhD etc.) |
| | Professional Degree (MBA/Law/Medical etc.) |
| | Other |

What is your occupation?

| | |
|---|---|
| | Student |
| | Employed in government organization |
| | Employed in private organization |
| | Self Employed |
| | Retired |
| | Housewife |
| | Other |

Which city do you live in?

| |
|---|
| |

What is the postal code of the area you live in?

| |
|---|
| |

.
